# The magnitude of precancerous cervical lesions and its associated factors among women screened for cervical cancer at a referral center in southern Ethiopia, 2021: a cross-sectional study

**DOI:** 10.3389/fgwh.2023.1187916

**Published:** 2023-08-16

**Authors:** Lidiya Tenkir, Abyalew Mamuye, Wegene Jemebere, Tomas Yeheyis

**Affiliations:** ^1^ School of Medicine, College of Medicine and Health Sciences, Hawassa University, Hawassa, Ethiopia; ^2^ School of Nursing, College of Medicine and Health Sciences, Hawassa University, Hawassa, Ethiopia

**Keywords:** magnitude, precancerous cervical lesion, cervical cancer, associated factors, southern Ethiopia

## Abstract

**Background:**

Cervical cancer is the most prevalent gynecologic cancer in women and the second leading cause of cancer death worldwide. Africa's southern, eastern, and western regions have the highest rates of cervical cancer. Cervical cancer is treatable and curable when detected early, but it is nonetheless fatal in low- and middle-income (LMIC) nations where screening and early detection are not widely accessible.

**Objective:**

The objective of this study is to assess the magnitude of precancerous cervical lesions and their associated factor among women screened for cervical cancer at a referral center in southern Ethiopia, in 2021.

**Methods:**

A retrospective institution-based cross-sectional study was conducted from February 21 to April 14, 2022, among randomly selected 372 records of women screened for cervical cancer at Hawassa University Specialized Hospital. Data were extracted from clients' charts using a data extraction checklist. Statistical Package for Social Sciences version 26 and logistic regression analysis were applied to determine the association between dependent and independent variables, and significance was declared at *p*-value <0.05.

**Results:**

The magnitude of abnormal cervical dysplasia in this study was 18.3% of which 14% were precancerous cervical lesions and 4.3% were cervical cancer. Early coitarche: Adjusted odds ratio (AOR) = 5.6 [95% (confidence interval) CI = 1.87–16.78], having more than one sexual partner: AOR = 2.6 [95% CI = 1.2–5.68], being HIV positive: AOR = 3.56 [95% CI = 1.53–8.29], and having sexually transmitted infections: AOR = 4.64 [95% CI = 2.08–10.35] were independent predictors of precancerous cervical lesions.

**Conclusion:**

The magnitude of precancerous cervical lesions in the study setting is 18% and the magnitude of cervical cancer is 14%, which is higher than the pooled prevalence of precancerous cervical lesions in Ethiopia. Having multiple sexual partners, being HIV positive, having sexual intercourse before 21 years, and new STI diagnosis was independently associated with abnormal precancerous cervical lesions.

## Introduction

Columnar cells undergo modifications in a precancerous stage of the cervix, increasing their propensity to develop into cancer. Although this stage has not yet developed into cancer, if it is not treated quickly, it is quite likely to do so in 10 years or more (1, 2). The area of the cervix that contains epithelium that has undergone a squamous metaplastic transition is known as the transformation zone. There is an area of squamous metaplasia between the ectocervix and the squamocolumnar junction. The majority of anomalies are assumed to start in this region (3).

Cervical cancer is the most common gynecologic cancer in women. The human papillomavirus is the primary cause of the majority of these malignancies, while other hosts and behavioral variables also play a role in the development of the tumors after the first infection. Serology is also inconsistent and unable to differentiate between past and present infections. Consequently, the only way to be certain of a diagnosis is through the direct detection of HPV DNA or cytologic examination. The absence of routine pap smear screening is the biggest risk factor for cervical cancer (2).

Sub-Saharan Africa has the highest global incidence and mortality rates for cervical cancer, with rates particularly high in Eastern Africa (Malawi has the highest incidence and mortality rate in the world), Southern Africa, and Middle Africa. In Northern America, Australia/New Zealand, and Western Asia (Saudi Arabia and Iraq), mortality rates can vary by up to 18 times, while incidence rates are 7–10 times lower (4, 5).

Africa's southern, eastern, and western regions have the greatest rates of cervical cancer. Cervical cancer is still fatal in low- and middle-income countries (LMIC), where screening and early detection are not easily accessible although it is a preventable illness that can be cured when discovered early. Almost 90% of cervical cancer fatalities occur in LMICs. LMICs frequently struggle with a lack of resources and funds to offer proper treatment (4–6).

Cervical cancer is thought to be a concern for 29 million Ethiopian women, with 7,095 new cases and 4,752 fatalities reported annually. Cervical cancer is the main cause of cancer-related deaths among women aged 15–44 in the nation and the second most common malignancy among all women (7, 8).

By 2020, the Federal Ministry of Health in Ethiopia aimed to have at least 80% of the eligible target groups screened and treated for cases of pre-invasive cervical cancer. Yet, a community-based cross-sectional survey of nine districts and two city administrations in Ethiopia (Addis Ababa and Dire Dawa) reveals a shockingly low percentage of cervical screening (2.9%), and only 10% of patients had visited the cancer center in the early stages of their illness (7, 8).

Though several studies concentrating on north and central Ethiopia are conducted, most of these studies are conducted focusing on women with HIV. Besides assessing all women screened for cervical cancer, this study is conducted in the biggest referral center in southern Ethiopia which encompasses not only urban but also women from rural and pastoral areas of the south. Therefore, this study aimed to determine the magnitude of precancerous cervical lesions and their predictors among women screened for cervical cancer in Hawassa University Comprehensive Specialized Hospital Southern Ethiopia.

After identifying the prevalence of precancerous cervical lesions and associated factors in the study area, this study aims to provide the finding of the study to Sidama, SNNPR, and Oromia regional health bureaus to enact strategies helpful for reducing contributors of the infection.

## Methods and materials

### Study setting

The Sidama area, one of the three densely populated coffee-producing regions in the nation, has Hawassa as its capital. HUCSH is a teaching hospital in Hawassa that has been providing health services since 2006. It provides services for more than 20 million people from all over southern Ethiopia. Currently, it has over 450 beds and cares for clients in a broad range of services to over 90,200 outpatients, 181,116 inpatients, and 1,092 emergency cases annually. The hospital gives cervical cancer screening services with VIA for women aged 30–49 years.

### Study period and study design

A retrospective institution-based cross-sectional method was employed from February 21 to April 14, 2022, to determine the magnitude of cervical cancer and its associated factors among women screened for cervical cancer at HUCSH in 2021.

### Study population

Medical records of all women who were screened for cervical cancer in the gynecologic department of HUCSH in 2021.

### Eligibility criteria

Medical records of women who were screened for cervical cancer available during the study period with complete information were recruited into the study including date of VIA examination, HIV status, and result of the VIA examination.

### Sample size determination

From the total number of 454 women screened for cervical cancer in 2021 at Hawassa University Comprehensive Specialized Hospital, 42 medical records were unavailable at the record center and 40 had incomplete information which resulted in the reduction of the sample size of the study to 372 charts of women screened for cervical cancer.

### Variables of the study

#### Dependent variables

The prevalence of precancerous cervical lesions.

#### Independent variables

Socio-demographic characteristics (age, marital status, occupation, and educational status), sexual activity (number of sexual partners), age at first sexual intercourse, parity, status of HIV infection, STI status, use of contraceptives, and history of smoking.

### Operational definition

#### Positive for cervical cancer

Women who have been screened using visual inspection with acetic acid (VIA) and tested positive.

### Data collection

Data was collected by investigators from the HUCSH pre-cervical cancer screening unit of the client assessment form and the registry book as a form of the checklist. It contains means of identification (age, educational status, and other sociodemographic statuses like marital status and occupation), reproductive history (marital status, parity, contraceptive use, age at first intercourse, and pregnancy), menstrual bleeding pattern (regularity and postcoital bleeding), risk factors (history of smoking, number of sexual partners, previous abnormal pap smear, chronic corticosteroid use, and HIV status), examination result, VIA result, and intervention done, if any.

### Data processing and analysis

Statistical Package for Social Science (SPSS) Version 26 was used to code, enter, clean up, and update the data before analysis. The statistical relationship between each independent variable and the result variable was examined using a logistic regression model. Variables with a *p*-value of 0.25 or less were added to the multivariable logistic regression model. Tables and texts were used to display the results. Using adjusted odds ratios (AOR) with a 95% confidence interval, the strength of associations was evaluated, and significance was determined at a *p*-value of 0.05.

### Data quality control

Training and orientation were provided for data collectors and supervisors for 2 days on the objective of the study, confidentiality of information, and data collection tools. Day-to-day supervision was carried out by the principal investigator and trained supervisor for the entire length of the data collection period.

Using the variance inflation factor and standard error, multi-collinearity was examined to determine whether there was a linear correlation between the independent variables. The multivariate analysis excluded variables having a standard error of >2 and a variance inflation factor >10 from consideration. To assess the model's fitness, a Hosmer-Lemeshow test was conducted. The test yielded a value >0.05, and the fit was assumed.

### Ethical considerations

Ethical approval was obtained from the institutional review board (IRB) of Hawassa University College of Medicine and Health Sciences. A formal letter was written to Hawassa University's comprehensive specialized hospital to access data of women screened for cervical cancer during the study period. Data were kept confidential, the anonymity of the respondents was assured, and data were used only for research purposes.

## Results

### Sociodemographic characteristics of the respondents

Out of 454 women screened for a precancerous lesion at HUCSH by VIA in the year 2021, a total of 372 medical records were reviewed in this study. The mean age of the participants was 38.68 (SD ± 9.07) years. Nearly 46.8% of participants had an educational level of elementary school and the majority of the participants were married (317) (85.2%) (Table 1).

**Table 1:** Socio-demographic characteristics of women screened for cervical cancer at HUCSH, 2021.

Variables (*n* = 372)		Frequency	Percent (%)
Age	21–30	77	20.69
	31–40	139	37.37
	41–50	116	31.19
	51–60	40	10.75
Marital status	Single	12	3.2
	Married	317	85.2
	Divorced	29	7.8
	Widowed	14	3.8
Educational status	No formal education	124	33.3
	Elementary school	174	46.8
	High school	55	14.8
	Higher education	19	5.1

### Reproductive history and related variables of the respondents

The participants' average starting age for sexual activity was 19.64 years (SD: 2.95). The majority of participants, 363 (97.6%), had at least one child, and 193 (51.8%) of them had more than three. A history of STI was present in nearly one-fifth of the individuals [73 (19.6%)], and 82 (22.04%) had several partners. More than a fourth-fifth of the participants 308 (82.79%) had been tested for HIV and forty-seven (12.6%) of them were reactive of which 44 (93.6%) were on highly active antiretroviral therapy (HAART) (Table 2).

**Table 2:** Reproductive health history and related characteristics of women screened for cervical cancer at HUCSH, 2021.

Variables (*n* = 372)		Frequency	Percent (%)
Age at first sexual intercourse	<17	45	13.7
	17–21	200	60.6
	>21	85	25.75
Parity	0	9	2.4
	1–3	200	53.76
	4–6	168	45.16
	>6	25	6.72
Number of sexual partners	1	290	77.95
	>1	82	22.05
History of STI	Yes	73	19.6
	No	299	80.4
New STI case	Yes	53	14.2
	No	319	85.8
Menstruation cycle	Regular	127	34.1
	Irregular	141	37.9
	Menopause	104	28.0
Current contraception	Yes	118	31.7
	No	254	68.3
Smoking history	Yes	6	1.6
	No	366	98.4
HIV status	Positive	47	12.6
	Negative	261	70.2
	No screened	64	17.2

### The magnitude of abnormal cervical lesions

**Figure 1:**
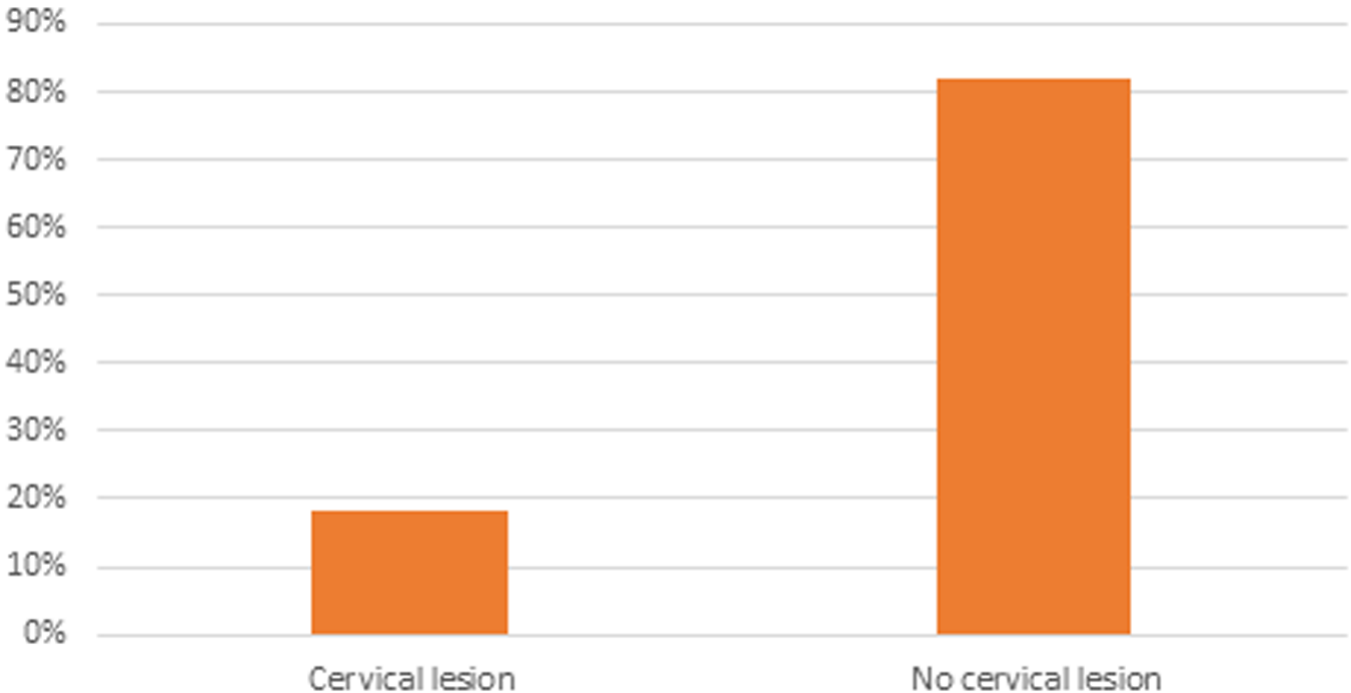
The magnitude of precancerous cervical lesions by VIA screening among women screened for cervical cancer at HUCSH, 2021.

Among 372 women's medical records, 18.3% of participants had abnormal cervical lesions, of which 14% were found to be positive and 4.3% had lesions suspected to be cervical cancer (Figure 1).

Of the 372 women screened for cervical cancer in the duration of the study, 96.8% had a normal pelvic exam, 86.3% were screened for the first time, 4.6% had a suspicious lesion upon examination, 14% had a positive VIA result, and 13.7% received cryotherapy (Table 3).

**Table 3:** Screening result of women screened for cervical cancer at HUCSH, 2021.

Variable		Frequency	Percent (%)
Pelvic exam	Normal	360	96.82
	Abnormal	12	3.18
Suspicious for cervical cancer	Yes	17	4.6
	No	355	95.4
SCJ was completely seen	Yes	337	90.6
	No	35	9.4
Type of screening visit	1st time	321	86.3
	Re-screening	50	13.7
VIA result	Negative	304	81.7
	Positive	52	14

### Factors associated with precancerous cervical lesion

The multivariable logistic regression analysis showed that early age coitarche, having more than one sexual partner, being HIV positive, and new STI history were independently associated with abnormal cervical lesions. The odds of the abnormal cervical lesion were 5.6 times higher among women whose age at first sexual intercourse was <21 years old, AOR = 5.6 [95% CI = 1.87–16.78]. Women who had more than one sexual partner were 2.6 times more likely than women who had only one partner to acquire abnormal cervical lesions, AOR = 2.6 [95% CI = 1.2–5.68]. Those who were HIV+ had a higher chance of developing atypical cervical lesions, AOR = 3.56 [95% CI = 1.53–8.29]. The odds of the abnormal cervical lesion were 4.64 times higher among women who had new sexually transmitted infections (STI) AOR = 4.64 [95% CI = 2.08–10.35] (Table 4).

**Table 4:** Bivariable and multivariable logistic regression analysis of factors associated with precancerous cervical lesions among women screened for cervical cancer at HUCSH, 2021.

Variable		VIA result		COR (95% CI)	AOR (95% CI)
		Negative	Positive		
Coitarche	>21	123	4	1	1
	<21	181	48	8.15 (2.86–23.2)	5.6 (1.87–16.8)*
Number of sexual partners	1	250	30	1	1
	>1	54	22	3.39 (1.82–6.33)	2.62 (1.21–5.68)*
HIV status	Negative	223	29	1	1
	Positive	26	16	4.73 (2.27–9.85)	3.56 (1.53–8.29)*
New STI	No	274	30	1	1
	Yes	30	22	6.7 (3.43–13.04)	4.64 (2.08–10.3)*
Age bicat^a^	>30	242	38	1	1
	<30	62	4	1.438 (0.73–2.82)	1.469 (0.71–3.02)
History of STI^a^	No	243	44	1	1
	Yes	61	8	0.724 (0.324–1.618)	0.66 (0.29–1.52)
History of smoking^a^	No	296	40	1	1
	Yes	8	12	6.143 (1.205–31.307)	6.799 (1.31–35.227)
Current contraception use^a^	No	206	34	1	1
	Yes	98	18	1.113 (0.599–2.068)	1.050 (.53–2.04)

^a^ The variables which were adjusted to get the odds ratios were smoking history, history of STI, contraception use, and age.

*p value <0.05.

## Discussion

This study assessed the prevalence of precancerous cervical lesions and associated factors among women screened for cervical cancer at HUCSH in 2021. In this study, women were screened with visual inspection with acetic acid (VIA) for precancerous cervical lesions. Because VIA is less expensive and uses resources that are readily available locally, it is preferred in low-resource nations like Ethiopia. In this study, 18% had a suspicious precancerous cervical lesion, 14% were positive for precancerous cervical lesions tested by visual inspection with acetic acid, and 4.3% had lesions suspected to be cervical cancer.

The magnitude of precancerous cervical lesions in this study (14%) is consistent with the finding of a study in the Amhara region of northern Ethiopia (13.1%), and a systematic review and meta-analysis pooled the prevalence of cervical precancerous lesions among women in Ethiopia (13.4%) (9, 10).

Compared to other previous studies, the finding of the study is less than what was reported in a study conducted at Gandhi memorial hospital, Ethiopia, (23.5%) and a study conducted among women greater than 30 years of age in southern Ethiopia (27.7%) (11, 12). The discrepancy between our study and the one conducted in Addis Ababa can be due to the nature of the center in Addis Ababa where the study was conducted. Which is one of the main referral centers in the country for screening for cervical cancer for suspected cases from different parts of the country. On the other hand, the discrepancy with the findings of the second study can be attributed to the fact that the study was conducted among women older than 30 years of age in the urban areas of southern Ethiopia, whereas our study encompasses women from both rural and pastoral areas of the region.

On the other hand, this study's outcome is higher than studies from Cameroon (3.33%), Ghana (3.7%), India (1%), and Northeast Ethiopia (6.9%) (3, 13–15). This difference in the magnitude of the condition can be attributed to the fact that these studies are done among a large population in the community, including women not screened for cervical cancer, and over extended years.

In this study, women who had more than one sexual partner were found to have a 2.6 times higher risk for cervical precancerous lesion than those who reported having one sexual partner. This finding is supported by a result from a study conducted in Addis Ababa, Ethiopia, Northeast Ethiopia, southern Ethiopia, Addis Ababa, and other African countries (11, 12, 15–20). Sexual contact is the primary method of transmission for the human papillomavirus, which causes precancerous cervical lesions. As with other sexually transmitted diseases, the risk of acquiring the virus increases with having sexual intercourse with multiple partners as it increases the chance of having more frequent and unprotected coitus (1, 2).

In the study, women who were HIV positive were found to have 3.56 times greater odds of having precancerous cervical lesions than women who were HIV negative. This finding is supported by a study on the global burden of the disease and a study in northeast Ethiopia which shows that the pooled risk of cervical cancer was increased by sixfold in women living with HIV (15, 21). HIV and cervical cancer are inextricably linked. HIV-positive women are commonly assumed to have more risky sexual behavior than their counterparts. The immune system is crucial for eliminating cancer cells and reducing their development and dissemination. HIV-infected women have impaired cell-mediated immunity that makes them more prone to the cancerous effect of HPV; this sequence of risks exposes them to having a precancerous cervical lesion more easily than HIV-negative women (2, 22).

The study found that women who started having sexual intercourse before the age of 21 had 5.6 times higher odds of acquiring precancerous cervical lesions than those whose first sexual intercourse was above the age of 21 years. This result is in line with research conducted in Addis Ababa, southern Ethiopia, and the northeast of Ethiopia (12, 15, 17, 23). Because of the biological tendency of the young cervix during adolescence that may be more sensitive to chronic HPV infection, first sexual contact at an early age exposes one to the early acquisition of the HPV virus (24). Having sex at a younger age increases the chance of having sex for longer years in life, which, in turn, can raise the risk of acquiring cervical cancer (25).

In this study, women having new STI cases were found to have 4.64 times higher odds of being VIA positive for cervical cancer than those who had no STI at the time of screening. This finding is consistent with a study conducted in northeast Ethiopia, Amhara region in northern Ethiopia, and Gojjam in northwest Ethiopia (10, 15, 20, 26). The most prevalent sexually transmitted infection is the human papillomavirus (HPV), of which two types—HPV-16 and HPV-18—are responsible for approximately 50% of high-grade cervical pre-cancers (27). According to studies, Chlamydia bacteria and other STIs enable HPV to multiply and survive in the cervix, which may raise the risk of cervical cancer (3, 28). Furthermore, the fact that STIs and the virus that causes cervical cancer share a common route of transmission and risk factors can be linked to the high prevalence of cervical cancer among women with STIs (2).

## Conclusion and recommendation

In conclusion, this study found that the prevalence of precancerous cervical lesions in this study is higher than the pooled prevalence of precancerous cervical lesions in Ethiopia. To lower the prevalence of abnormal cervical lesions in the study area, the Federal Ministry of Health should concentrate on the primary prevention strategy (HPV vaccine) at an earlier stage. Many sexual partners, HIV positivity, having had sex before the age of 21, and a recent STI diagnosis were all separately linked to atypical precancerous cervical lesions.

### Limitations of the study

Because our data is secondary, we were not able to find some of the medical records. Some information from the checklist which was important for our study was missing. Since it was an already prepared checklist and the participants could not be addressed at the time of the study, some additional associated factors were not assessed. Hence, the study can be generalized to women screened at this University hospital.

## Data Availability

The raw data supporting the conclusions of this article will be made available by the authors, without undue reservation.

## References

[1] FMOH. Guideline for Cervical Cancer Prevention and Control in Ethiopia (2015). Addis Ababa: Federal Democratic Republic of Ethiopia Ministry of Health. https://www.iccp-portal.org/system/files/plans/Guideline%20Eth%20Final.pdf (Accessed February 7, 2023).

[2] Hoffman B Schorge J Schaffer J Halvorson L Bradshaw K Cunningham F. Williams Gynecology. 2nd ed. McGraw-Hill Professional (2012). 1401 p.

[3] Nkfusai NC Mubah TM Yankam BM Tambe TA Cumber SN. Prevalence of precancerous cervical lesions in women attending Mezam Polyclinic Bamenda, Cameroon. Pan Afr Med J. (2019) 32:174. 10.11604/pamj.2019.32.174.16895 31303943 PMC6607283

[4] Sung H Ferlay J Siegel RL Laversanne M Soerjomataram I Jemal A Bray F. Global cancer statistics 2020: GLOBOCAN estimates of incidence and mortality worldwide for 36 cancers in 185 countries. CA Cancer J Clin. (2021) 71(3):209–49. 10.3322/caac.21660 33538338

[5] De Martel C Plummer M Vignat J Franceschi S. Worldwide burden of cancer attributable to HPV by site, country and HPV type. Int J Cancer. (2017) 141(4):664–70. 10.1002/ijc.30716 28369882 PMC5520228

[6] Burt LM McCormak M Lecuru F Kanyike DM Bvochora-Nsingo M Ndlovu N Cervix cancer in sub-Saharan Africa: an assessment of cervical cancer management. JCO Glob Oncol. (2021) 7:173–82. 10.1200/GO.20.00079 33529076 PMC8081497

[7] Assefa AA Astawesegn FH Eshetu B. Cervical cancer screening service utilization and associated factors among HIV positive women attending adult ART clinic in public health facilities, Hawassa town, Ethiopia: a cross-sectional study. BMC Health Serv Res. (2019) 19:1. 10.1186/s12913-018-3827-x 31744548 PMC6862783

[8] Gemeda EY Kare BB Negera DG Bona LG Derese BD Akale NB Prevalence and predictor of cervical cancer screening service uptake among women aged 25 years and above in Sidama zone, Southern Ethiopia, using health belief model. Cancer Control. (2020) 27(1):1073274820954460. 10.1177/1073274820954460 32951445 PMC7791476

[9] Zena D Elfu B Mulatu K. Prevalence and associated factors of precancerous cervical lesions among women in Ethiopia: a systematic review and meta-analysis. Ethiop J Health Sci. (2021) 31(1):189–200. 34158766 10.4314/ejhs.v31i1.21 PMC8188114

[10] Temesgen MM Alemu T Shiferaw B Legesse S Zeru T Haile M Prevalence of oncogenic human papillomavirus (HPV 16/18) infection, cervical lesions and its associated factors among women aged 21–49 years in Amhara region, northern Ethiopia. Plos One. (2021) 16(3) e0248949. 33760866 10.1371/journal.pone.0248949 PMC7990306

[11] Mekuria M Edosa K Endashaw M Bala ET Chaka EE Deriba BS Prevalence of cervical cancer and associated factors among women who attended cervical cancer screening center at Gahandi memorial hospital, Ethiopia. Cancer Inform. (2021) 20:11769351211068431. 10.1177/11769351211068431 34992337 PMC8725021

[12] Teka T Kote M Kejela G Getachew T. Magnitude and factors associated with precervical cancer among screened women in southern Ethiopia. Adv Public Health. (2019) 2019. 10.1155/2019/5049752

[13] Donkoh ET Agyemang-Yeboah F Asmah RH Wiredu EK. Prevalence of cervical cancer and pre-cancerous lesions among unscreened women in Kumasi, Ghana. Medicine (Baltimore). (2019) 98(13):e14600. 10.1097/MD.0000000000014600 30921178 PMC6456016

[14] Kalavathy MC Mathew A Krishna KJ Saritha VN Sujathan K. Risk factors and prevalence of cervical squamous intraepithelial lesions among women in south India: a community-based cross-sectional study. Indian J Cancer. (2022) 59(1):95–100. 10.4103/ijc.IJC_699_19 33753607

[15] Temesgen K Workie A Dilnessa T Abate M. Proportions of pre-cancerous cervical lesions and its associated factors among women clients in the age group of 30−49yrs in gynecology ward of dessie referral hospital and FGAE, north-east Ethiopia, 2016. J Cancer Tumor Int. (2019) 9(2):1–15. 10.9734/jcti/2019/v9i230105

[16] World Health Organization and International Agency for Research on Cancer. Prevention of cervical cancer through screening using visual inspection with acetic acid (VIA) and treatment with cryotherapy. A demonstration project in six African countries: Malawi, Madagascar, Nigeria, Uganda, the United Republic of Tanzania, and Zambia (2012). Available at: https://www.who.int/publications/i/item/9789241503860 (Accessed February 5, 2023).

[17] Teame H Addissie A Ayele W Hirpa S Gebremariam A Gebreheat G Factors associated with cervical precancerous lesions among women screened for cervical cancer in Addis Ababa, Ethiopia: a case-control study. PloS One. (2018) 13(1):e0191506. 10.1371/journal.pone.0191506 29352278 PMC5774809

[18] Weldegebreal F Worku T. Precancerous cervical lesion among HIV-positive women in sub-Saharan Africa: a systematic review and meta-analysis. Cancer Control. (2019) 26(1):1073274819845872. 10.1177/1073274819845872 31043067 PMC6572896

[19] Ali-Risasi C Verdonck K Padalko E Vanden Broeck D Praet M. Prevalence and risk factors for cancer of the uterine cervix among women living in Kinshasa, the democratic republic of the Congo: a cross-sectional study. Infect Agents Cancer. (2015) 10:1–11. 10.1186/s13027-015-0015-z PMC4502934 26180542

[20] Jolly PE Mthethwa-Hleta S Padilla LA Pettis J Winston S Akinyemiju TF Screening, prevalence, and risk factors for cervical lesions among HIV positive and HIV negative women in Swaziland. BMC Public Health. (2017) 17(1):1–8. 10.1186/s12889-017-4120-3 28222714 PMC5320649

[21] Stelzle D Tanaka LF Lee KK Khalil AI Baussano I Shah AS Estimates of the global burden of cervical cancer associated with HIV. Lancet Glob Health. (2021) 9(2):e161–9. 10.1016/S2214-109X(20)30459-9 33212031 PMC7815633

[22] Choudhury SA Choudhury NA Humphrey AD Berthaud V Ladson G Tucker VA Higher prevalence of human papillomavirus-related cervical precancerous abnormalities in HIV-infected compared to HIV-uninfected women. J Natl Med Assoc. (2016) 108(1):19–23. 10.1016/j.jnma.2015.12.003 26928484 PMC10767705

[23] Makuza JD Nsanzimana S Muhimpundu MA Pace LE Ntaganira J Riedel DJ. Prevalence and risk factors for cervical cancer and pre-cancerous lesions in Rwanda. Pan Afr Med J. (2015) 22:1. 10.11604/pamj.2015.22.26.7116 PMC4662515 26664527

[24] Abebe M Eshetie S Tessema B. Prevalence of sexually transmitted infections among cervical cancer suspected women at university of Gondar comprehensive specialized hospital, north-west Ethiopia. BMC Infect Dis. (2021) 21(1):1–7. 10.1186/s12879-021-06074-y 33888090 PMC8063310

[25] Liu ZC Liu WD Liu YH Ye XH Chen SD. Multiple sexual partners as a potential independent risk factor for cervical cancer: a meta-analysis of epidemiological studies. Asian Pac J Cancer Prev. (2015) 16(9):3893–900. 10.7314/APJCP.2015.16.9.3893 25987056

[26] Getinet M Taye M Ayinalem A Gitie M. Precancerous lesions of the cervix and associated factors among women of east Gojjam, northwest Ethiopia, 2020. Cancer Manag Res. (2021) 2021:9401–10. 10.2147/CMAR.S338177 PMC8721437 35002317

[27] UN AIDS. HIV and cervical cancer. United Nations. (2022). Available at: https://www.unaids.org/en/resources/documents/2022/HIV-and-cervical-cancer (Accessed at 6 February 2023).

[28] Quinn TC Wawer MJ Sewankambo N Serwadda D Li C Wabwire-Mangen F Viral load and heterosexual transmission of human immunodeficiency virus type 1. N Engl J Med. (2000) 342(13):921–9. 10.1056/NEJM200003303421303 10738050

